# Marine-Derived Yaequinolone Derivative CHNQD-02792 Suppresses Colorectal Cancer Cell Proliferation and Induces Apoptosis via MAPK Pathway Modulation

**DOI:** 10.3390/md23040136

**Published:** 2025-03-21

**Authors:** Jia-Qi Kang, Tian-Yi Zhou, Wen-Hui Wang, Mei-Yan Wei, Chang-Lun Shao

**Affiliations:** Key Laboratory of Marine Drugs, The Ministry of Education of China, School of Medicine and Pharmacy, Ocean University of China, Qingdao 266003, China; kangjiaqi1210@163.com (J.-Q.K.); zty17342090270@163.com (T.-Y.Z.); wwenhui163@163.com (W.-H.W.); mywei95@126.com (M.-Y.W.)

**Keywords:** yaequinolone derivative, colorectal cancer, cycle block, apoptosis, proliferation inhibition

## Abstract

Colorectal cancer is currently the third most common malignancy, and the toxic side effects of clinical therapeutic drugs often influence treatment outcomes. Marine-derived quinolone alkaloids exhibit various biological activities and are particularly notable for their antitumor properties. Compounds **1**–**13** were semi-synthesized based on 4′-desmethoxyyaequinolone J1, which is a 4-phenyl derivative of the natural quinolone alkaloid yaequinolone J1 and was isolated from *Penicillium* sp. FKI-2140. This study is the first to investigate the antitumor activity of **1**–**13** in colorectal cancer cells through proliferation, clonality, apoptosis, cell cycle, and MAPK signaling pathway. Cytotoxicity screening against seven colorectal cancer cell lines revealed that CHNQD-02792 (**13**) had the most sensitivity to HT-29 cells (IC_50_ = 4.5 μM), far exceeding positive control 5-fluorouracil (IC_50_ = 15.58 μM). The plate cloning assay revealed that CHNQD-02792 completely inhibited the growth of HT-29 cells at the concentration of 9 μM. CHNQD-02792 (4.5 μM) inhibited CDK1 expression and triggered G2/M phase arrest in HT-29 cells. Mechanistic analysis revealed that CHNQD-02792 induced apoptosis by suppressing the anti-apoptotic protein Bcl-2 and upregulating the pro-apoptotic proteins Caspase-3 and Bax. Furthermore, CHNQD-02792 inhibited ERK and JNK phosphorylation and thus highlighted its regulatory role in MAPK signaling. These findings suggest that CHNQD-02792 exerts cytotoxic effects on HT-29 cells via dual mechanisms: inducing G2/M arrest and apoptosis while regulating MAPK signaling through ERK/JNK dephosphorylation. This study demonstrates the dual targeting of CHNQD-02792 against tumor cell proliferation and survival pathways, providing a foundation for further development of anti-colorectal cancer drugs.

## 1. Introduction

Cancer is a major global public health challenge, ranking as the first or second leading cause of death among individuals under 70 in most countries [1,2,3], with colorectal cancer being particularly prevalent [4]. In 2020, an estimated 1.93 million colorectal cancer cases were reported globally, with an age-standardized mortality rate (ASMR) of 19.5 per 100,000, ranking fourth among major malignant tumors [5]. Colorectal cancer is projected to cause around 930,000 deaths, with an ASMR of 9.0 per 100,000, ranking third among major malignant tumors worldwide [6,7,8]. Primary prevention remains essential for reducing the global burden of colorectal cancer [9]. However, due to the high cost of colonoscopy and limited access to diagnostic and treatment services in many countries, the majority of colorectal cancer patients are diagnosed at advanced or metastatic stages [10,11]. Patients with advanced or metastatic colorectal cancer (mCRC) primarily receive systemic therapies [12,13,14]. The toxic side effects of current anti-colorectal cancer drugs pose significant challenges in clinical management [15]. Conventional chemotherapeutic agents (e.g., fluorouracil and oxaliplatin) frequently cause myelosuppression, neurotoxicity, and gastrointestinal injuries owing to their non-selective cytotoxicity [16,17], whereas targeted therapies such as anti-EGFR monoclonal antibodies may induce skin toxicity and hypomagnesemia [18]. Additionally, immune checkpoint inhibitors (e.g., PD-1 inhibitors) are associated with autoimmune complications like enteritis [19]. These adverse events not only deteriorate patients’ quality of life but also often necessitate dose reduction or treatment discontinuation, ultimately undermining clinical outcomes [20]. Consequently, prioritizing the development and selection of safer, highly targeted anticancer agents is imperative to enhance therapeutic precision and long-term survival.

**Figure 1:**
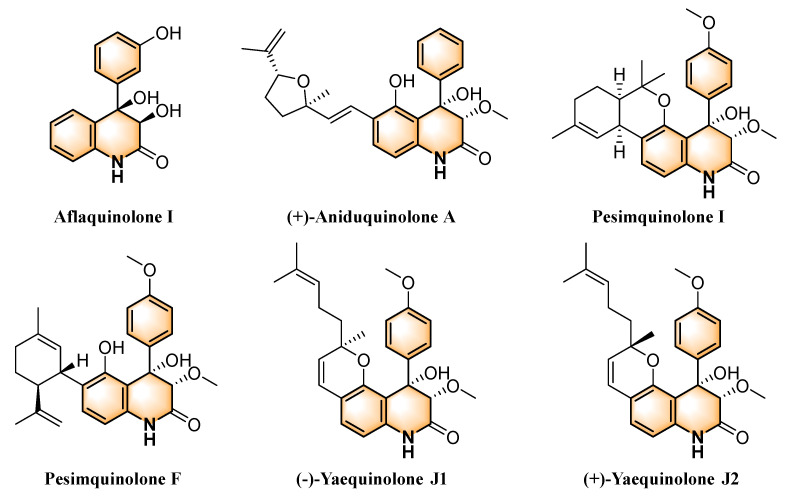
(−)-Yaequinolone J1, (+)-Yaequinolone J2, and representatives of the potential antitumor quinolone alkaloids.

Marine ecosystems are a rich source of bioactive compounds, and approximately 56% of these compounds exhibit antitumor properties [21]. These compounds exhibit a wide range of remarkable biological activities, including antitumor [22,23,24], antiviral [25], antimicrobial [26,27], and antifouling [26,28] effects. Notably, over the past 20 years, structurally unique 3,4-dioxo-5-hydroxy-4-aryl-quinolin-2(1H)-one alkaloids have been extensively identified in marine-derived fungi *Aspergillus* sp. and *Penicillium* sp., highlighting their potential as novel anticancer agents [29]. Recent studies have highlighted several representative compounds with potent antitumor and anti-inflammatory activities, including Aflaquinolone I [30], (+)-Aniduquinolone A [31], Pesimquinolone I [24], and Pesimquinolone F (Figure 1), which collectively underscore the therapeutic potential of this structural class. Quinolones exert antitumor effects through multiple mechanisms, including topoisomerases inhibition [32] and modulation of protein kinase signaling pathways [33,34]. These compounds offer new targets and insights for antitumor drug development. In-depth studies of their mechanisms of action may reveal additional potential targets, laying the foundation for novel anticancer therapies.

In our previous study, we designed and semi-synthesized a series of yaequinolone-like alkaloids using yaequinolones J1 and J2 [35], the natural racemic products with anti-inflammatory activity, as template molecules (Figure 1). Compounds **1**–**13** exhibited anti-inflammatory activity, with **13** significantly inhibiting NO release at 10 nM and IL-6 level at 50 nM in LPS-induced RAW264.7 cells. Given the remarkable activity of **13**, we named it CHNQD-02792. Chronic inflammation plays a critical role in cancer development and progression by accelerating tumor cell proliferation and metastasis [36]. It induces genomic instability by releasing pro-inflammatory cytokines and reactive oxygen species while fostering an immunosuppressive microenvironment [37]. Inhibition of inflammation-related pathways, including MAPK, has been shown to suppress pro-cancer responses and tumor growth [38]. In this study, we investigated the effects of CHNQD-02792 with anti-inflammatory properties on seven colorectal cancer cell lines and further explored the mechanisms underlying its inhibition of tumor cell growth. We first identified that CHNQD-02792 exhibits significant antitumor activity through screening and subsequently investigated its antitumor mechanisms in HT-29 cells, focusing on cell cycle progression and apoptosis.

## 2. Results and Discussion

### 2.1. Selectivity of Quinolone Derivatives for Various Colorectal Cancer Cell Lines

**Figure 2:**
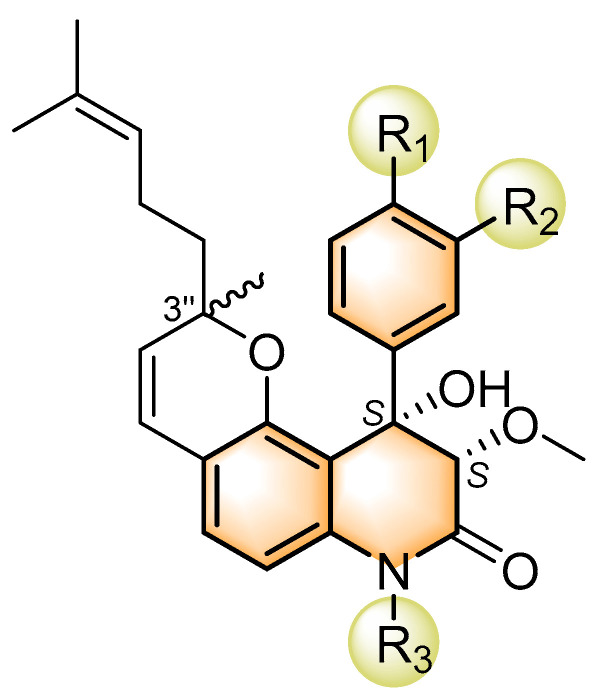
The structures of yaequinolone derivatives.

To evaluate the effects of yaequinolone derivatives on different colorectal cancer cell lines, we selected seven colorectal cancer cell lines for testing: CT-26, RKO, LoVo, HT-29, HCT-116, DLD-1, and LS-174T cells. The effects of yaequinolone derivatives on the proliferation of colorectal cancer cells were evaluated using the MTT assay. Figure 2 and Table 1 listed the 13 yaequinolone compounds that had better anti-inflammatory ability in our previous study [35]. The effects of these 13 compounds on the proliferation of seven colorectal cells were examined, and it was found that, except for compounds **4**, **7**, **12**, and **13**, the IC_50_ values of the other nine compounds on the inhibition of seven colorectal cancer cells were more than 50 μM.

**Figure 3:**
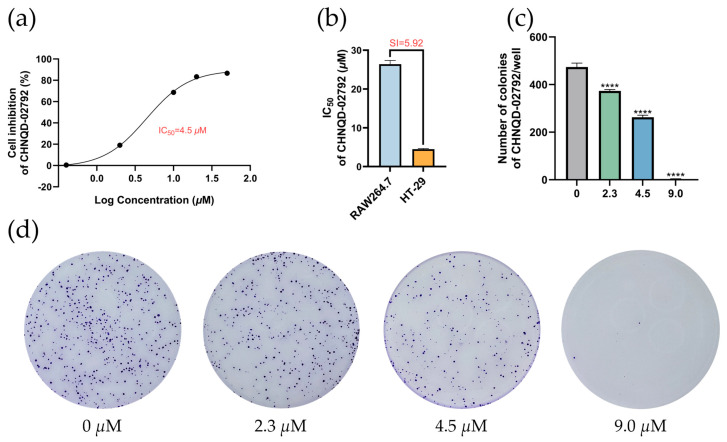
Effects of CHNQD-02792 on tumor cell proliferation and colony formation in HT-29 cells. (**a**) Cytotoxic effects of CHNQD-02792 on HT-29, LS-174T, and LoVo cells. (**b**) Cytotoxic effect of CHNQD-02792 on HT-29 cells. (**b**) The selectivity index (SI) of CHNQD-02792 for RAW264.7 and HT-29 cells. (**c**,**d**) Effect of CHNQD-02792 on clone formation. **** *p* < 0.0001 compared to the control group.

Compounds **4** and **7**, as well as compounds **12** and **13**, form two pairs of epimers, each containing three chiral centers, with only one differing in configuration. The IC_50_ values of compounds **4**, **7**, **12**, and **13** for inhibiting the growth of seven colorectal cancer cells are shown in Table 2. These four compounds had different inhibitory abilities on seven different cells. Specifically, compound **4** had the weakest inhibitory ability, exhibiting inhibition of RKO cells with an IC_50_ value of 48.3 μM. Compound **7** showed inhibitory ability against RKO cells and LoVo cells with IC_50_ values of 22.0 and 25.0 μM, respectively. Except for LoVo cells, compound **12** showed significant inhibition of the remaining six colorectal cancer cells, with inhibitory abilities ranging from strongest to weakest: LS-174T cells > DLD-1 cells > HCT-116 cells > CT-26 cells > RKO cells > HT-29 cells. Notably, CHNQD-02792 exhibited the strongest inhibitory ability of all compounds among the cells, and its IC_50_ for HT-29 inhibition was only 4.5 μM. As shown in Figure 3a, CHNQD-02792 did, in a dependent manner, inhibit the proliferation of HT-29 cells.

In addition, CHNQD-02792 also inhibited RKO cells, LoVo cells, and LS-174T cells, but the inhibitory capacity was much lower than that of HT-29 cells. As a result of preliminary experiments, we found that CHNQD-02792 had a strong anti-inflammatory effect on LPS-stimulated RAW264.7 cells. Therefore, to verify the difference between CHNQD-02792 on normal cells and cancer cells, we examined the inhibition of CHNQD-02792 on RAW264.7 cells. Surprisingly, CHNQD-02792 inhibited RAW264.7 with an IC_50_ of 27.20 μM, with a selectivity index (SI) of 5.92 ( Figure 3b). Therefore, we speculate that CHNQD-02792 exhibited selectivity in inhibiting tumor cells. Plate colony formation assays were conducted with CHNQD-02792, and the results are displayed in Figure 3c, d. The inhibition of HT-29 cell colony formation increased with higher concentrations of CHNQD-02792. These findings suggest that CHNQD-02792 effectively suppresses HT-29 cell proliferation.

### 2.2. CHNQD-02792 Induces HT-29 Cell Cycle Block in G2/M Phase

**Figure 4:**
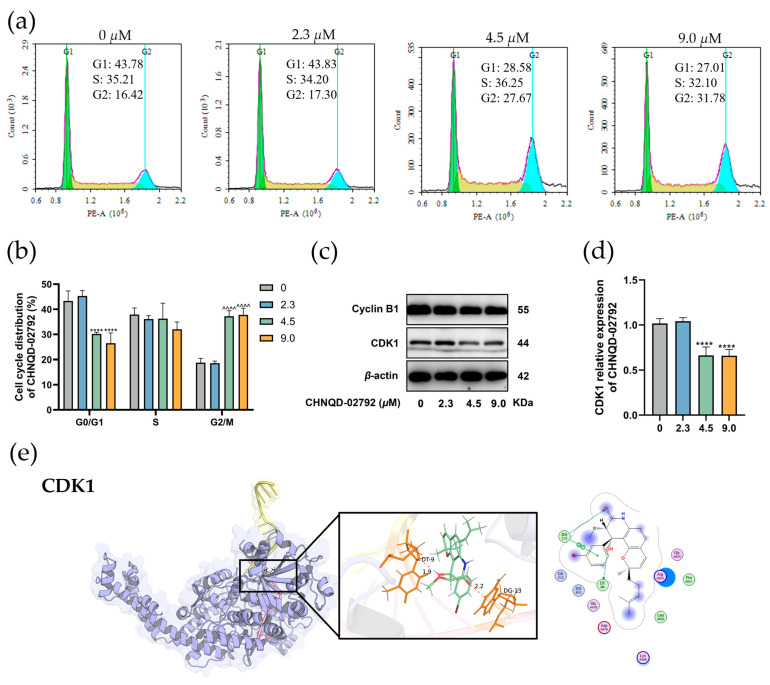
CHNQD-02792 induces HT-29 cell cycle block in the G2/M phase. (**a**,**b**) Flow cytometry was used to analyze the effects of different concentrations of CHNQD-02792 on the HT-29 cell cycle after 48 h of treatment. (**c**,**d**) Western blot analysis was performed to examine the effects of different concentrations of CHNQD-02792 on cell cycle-related proteins in HT-29 cells after 48 h of treatment. ^^^^/**** *p* < 0.0001 compared to the control group. (**e**) The molecular docking analysis of CHNQD-02792 binding to CDK1 (**e**) was performed using the MOE program, simulating their interaction at a molecular level; red dotted lines, H-bonds labeled with distances in Å.

Dysregulation of the cell cycle is a key factor in tumor development, and inducing cell cycle arrest can effectively inhibit tumor cell proliferation. To investigate the mechanism underlying the inhibition of HT-29 cell proliferation by CHNQD-02792. HT-29 cells were treated with varying concentrations of CHNQD-02792 for 48 h. Cell cycle changes were analyzed using flow cytometry, as shown in Figure 4. After treatment with CHNQD-02792, the proportion of cells in the G2/M phase increased from 16.42% to 31.78% in a dose-dependent manner compared with the control group. CHNQD-02792 promoted cell cycle entry into the G2/M phase and thus induced cell cycle arrest. Further study of the cell cycle distribution (Figure 4b) revealed that the higher the concentration of CHNQD-02792, the higher the proportion of cells in the G2/M phase, while the proportion of cells in the G0/G1 phase decreased. This suggested that CHNQD-02792 (4.5 μM) treatment induced accelerated cell entry into the M-phase or increased the proportion of cells in the G2/M-phase, thereby affecting cell division.

Cyclin B1 and CDK1 are key regulators of the G2/M phase, which is involved in the regulation of cell cycle progression. In Figure 4c,d, the protein expression levels of Cyclin B1 and CDK1 in HT-29 cells treated with different concentrations of CHNQD-02792 were analyzed by Western blot. The results showed that the expression levels of Cyclin B1 and CDK1 decreased with increasing concentrations of CHNQD-02792, which was particularly significant at high concentrations (**** *p* < 0.0001). This suggested that CHNQD-02792 affected cell cycle progression by inhibiting the expression of Cyclin B1 and CDK1.

Next, we performed molecular docking studies to determine the orientation of CHNQD-02792 bound in the active site of CDK1 using the MOE program as described. The analysis showed that CHNQD-02792 docked within the CDK1 with a binding energy of −8.043 kcal/mol and formed two hydrogen bonds with the DT 9 and DG 13 residues (Figure 4e). The compound also exhibited hydrophobic interactions with DG C13, DT B9, Leu A502, and Pro A501 residues of CDK1. These findings confirmed that CHNQD-02792 prevented cell entry into mitosis by binding to the ATP-binding pocket of CDK1, inhibiting CDK1 activity, and preventing the Cyclin B1-CDK1 complex from phosphorylating downstream targets. Consequently, HT-29 cells were arrested in the G2/M phase.

### 2.3. CHNQD-02792 Induces Apoptosis in HT-29 Cells

**Figure 5:**
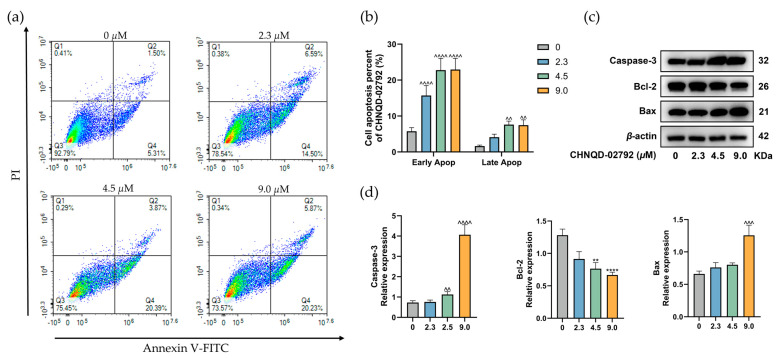
CHNQD-02792 induces apoptosis in HT-29 cells. (**a**,**b**) Annexin V-FITC/PI double staining and flow cytometry were used to evaluate the effects of different concentrations of CHNQD-02792 on HT-29 cell apoptosis after 48 h of treatment. (**c**,**d**) Western blot analysis was conducted to evaluate the effects of different concentrations of CHNQD-02792 on apoptosis-related proteins in HT-29 cells after 48 h of treatment. **/^^ *p* < 0.01, ^^^ *p* < 0.001, ****/^^^^ *p* < 0.0001 compared to the control group.

Cell cycle arrest has been reported to trigger apoptosis in the literature. To evaluate whether CHNQD-02792 induces apoptosis, HT-29 cells were treated with CHNQD-02792 and analyzed using Annexin V-FITC/PI double-staining flow cytometry (Figure 5a,b). Compared to the control group, the proportion of apoptotic cells increased from 6.81% to 26.10% after 48 h of treatment, with early apoptotic cells rising from 5.31% to 20.23%. By analyzing the percentage of early versus late apoptosis, it was found that an increase in the concentration of CHNQD-02792 led to a rise in the proportion of both early and late apoptotic cells. The percentage of late apoptotic cells was significantly higher in the CHNQD-02792 (9 μM) group.

Since CHNQD-02792 exhibited a pro-apoptotic effect, the expression levels of apoptosis-related proteins Caspase-3, Bcl-2, and Bax were further examined. Bcl-2 is an anti-apoptotic protein, while Bax is a pro-apoptotic protein, and Caspase-3 is an executioner enzyme in the apoptotic pathway. The results (Figure 5c,d) showed that with the increase in the concentration of CHNQD-02792, the protein expression of Caspase-3 significantly increased, the expression of Bcl-2 markedly decreased, and the expression of Bax also increased. Particularly in the high-concentration (9 μM) group, the protein expression levels of Caspase-3 and Bax reached their highest (^^^ *p* < 0.001, ^^^^ *p* < 0.0001), while the expression of Bcl-2 was at its lowest (**** *p* < 0.0001). This further demonstrated that CHNQD-02792 promotes apoptosis in colorectal cancer HT-29 cells by altering the balance between Bcl-2 and Bax, thereby activating Caspase-3.

### 2.4. Effect of CHNQD-02792 on MAPK Signaling Pathway

**Figure 6:**
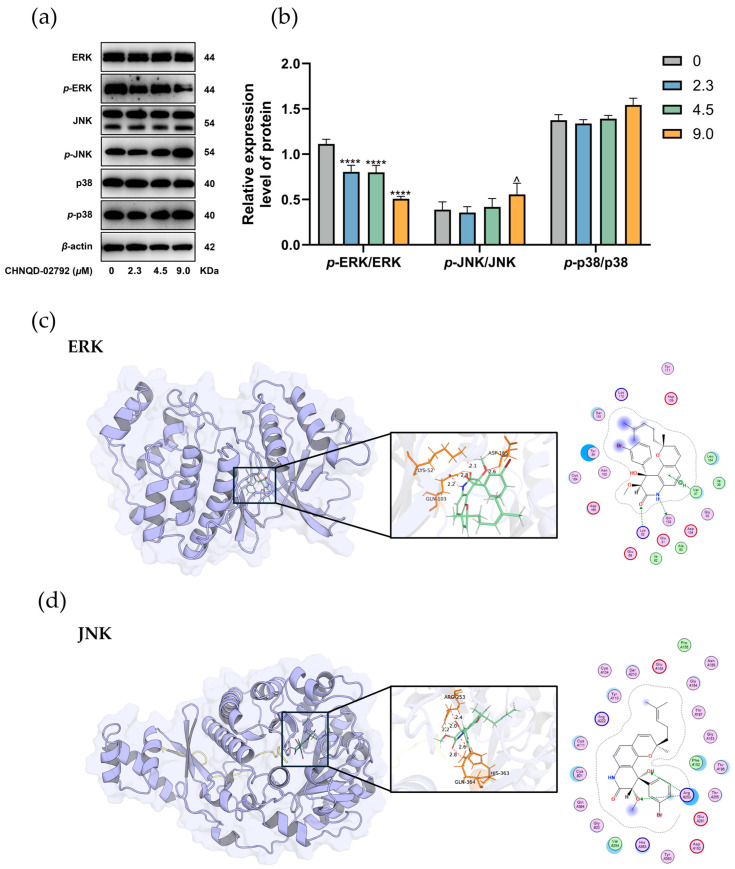
Effects of CHNQD-02792 on MAPK signaling pathway in HT-29 cells. (**a**,**b**) Western blot analysis was conducted to evaluate the effects of different concentrations of CHNQD-02792 on MAPK-related proteins in HT-29 cells after 48 h of treatment. ^ *p* < 0.05, **** *p* < 0.0001 compared to the control group. (**c**,**d**) The molecular docking analysis of CHNQD-02792 binding to ERK (**c**) and JNK (**d**) was performed using the MOE program, simulating their interaction at a molecular level; red dotted lines, H-bonds labeled with distances in Å.

Mitogen-activated protein kinases (MAPKs) are serine-threonine kinases activated by various extracellular stimuli, such as cytokines, neurotransmitters, hormones, cellular stress, and adhesion. MAPKs are key transmitters of signals from the cell surface to the nucleus. The MAPK pathway transmits, amplifies, and integrates signals from diverse stimuli, triggering physiological responses such as cell proliferation, differentiation, transformation, inflammation, and apoptosis. ERK, JNK, and p38 are key molecules in the MAPK signaling pathway. The effects of CHNQD-02792 on the phosphorylation of these molecules in HT-29 cells were examined. The results demonstrated that CHNQD-02792 inhibited the phosphorylation of ERK and increased the phosphorylation of JNK (Figure 6a,b).

We conducted molecular docking studies to determine the binding orientation of CHNQD-02792 in the active sites of ERK and JNK using the MOE program. The analysis showed that CHNQD-02792 docked into the ERK protein with a binding energy of −8.4076 kcal/mol, forming three hydrogen bonds with Lys 52, Asp 165, and Gln 103 (Figure 6c). The compound also exhibited hydrophobic interactions with Val 37, Ile 82, and Leu 154 of ERK. CNQD-02792 fits well into the ATP-binding pocket of JNK with a binding energy of −8.5296 kcal/mol, showing good binding affinity under physiological conditions. Additionally, CHNQD-02792 formed three hydrogen bonds with Arg 253, Gln 364, and His 363 of JNK (Figure 6d). It also exhibited hydrophobic interactions with Val A284, Phe A152, and Pro A156. CHNQD-02792 occupies the ATP-binding pocket, blocking JNK autophosphorylation (Thr 183/Tyr 185) and inhibiting downstream signaling. Overall, these findings confirm that CHNQD-02792 synergistically exerts antitumor effects through dual regulation of the MAPK pathway: inhibiting ERK phosphorylation (blocking pro-proliferative signaling) and activating JNK phosphorylation (inducing pro-apoptotic signaling).

### 2.5. Structure-Activity Relationship

**Figure 7:**
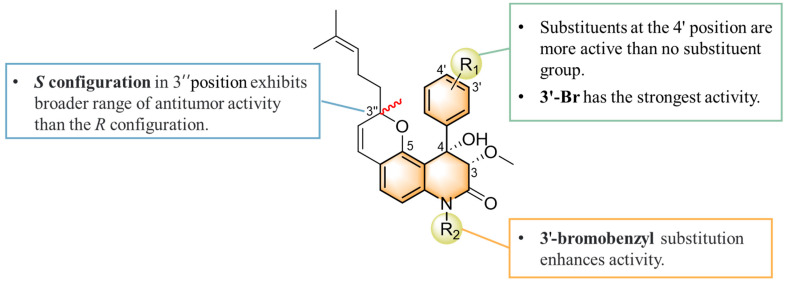
Structure-activity relationships of yaequinolone derivatives.

Combining our previously reported anti-inflammatory activity studies with the current antitumor cytotoxicity results, we found that the activity of yaequinolone derivatives is closely related to their absolute configuration (Figure 7). The 4-phenyl derivatives had good anti-inflammatory ability when substituted with no group (**1**, **2**), but no antitumor activity was found. The *S* configuration at the 3″ position (**7**, **12**) showed broader efficacy against colorectal cancer cell types compared to the *R* configuration. Substituents of 3′-Br (**12**, CHNQD-02792) on the 4-phenyl moiety greatly enhanced the activity. Phenyl substitution at the C4 position of 3′-Br (**12**, CHNQD-02792) was more effective than 4′-NO_2_ (**10**, **11**). The 3′-bromobenzyl substitution of the N-H position (**4**,**7**) was beneficial in enhancing the antitumor activity. The presence of an oxygen atom at the C5 position was also crucial for the antitumor activity of the compounds.

## 3. Materials and Methods

### 3.1. Main Instruments and Reagents

Roswell Park Memorial Institute (RPMI) 1640, Dulbecco’s Modified Eagle’s Medium (DMEM), Minimum Essential Medium (MEM), and Ham’s F-12K Nutrient Mixture (Ham’s F-12K) were all provided by GIBCO (Grand Island, NY, USA). Fetal Bovine Serum (FBS, PAN, Eidenbach, Germany). Primary antibodies against *β*-actin, CDK1, Cyclin B1, Bax, Bcl-2, Bax, Caspase-3, ERK, *p*-ERK, JNK, *p*-JNK, p38, and *p*-p38 were purchased from Cell Signaling Technologies (Danvers, MA, USA). Radio Immunoprecipitation Assay (RIPA) lysis buffer, phenylmethylsulfonyl fluoride (PMSF), phosphate-buffered saline (PBS), and protease inhibitors were supplied by Wuhan Saiweier Biotechnology Co., Ltd. (Wuhan, China).

### 3.2. Sample Information

In our previous work, compounds **1**–**13** were synthesized and characterized, with full synthetic protocols, structural validation data, purity analysis, and spectroscopic information reported in the established literature [35].

### 3.3. Cell Lines

Human colorectal adenocarcinoma cells HT-29, HCT-116, DLD-1, LS-174T, RKO, and LoVo, as well as mouse colorectal carcinoma cell CT-26, were purchased from the Chinese Academy of Sciences Shanghai Cell Bank.

### 3.4. Cell Culture

Cells stored at −80 °C were rapidly thawed in a 37 °C water bath, centrifuged to collect, and resuspended in a complete medium for culturing in a 37 °C, 5% CO_2_ incubator. Then, they were rinsed twice with PBS, digested with trypsin, and resuspended in a complete medium after detachment. HT-29, HCT-116, DLD-1, and CT-26 cells were cultured in RPMI-1640 medium supplemented with 10% FBS and 1% penicillin-streptomycin. LS-174T cells were cultured in DMEM-H medium, LoVo in Ham’s F-12K medium, and RKO in MEM.

### 3.5. MTT Assay

Cells in the logarithmic growth phase were seeded in 96-well plates at 4000 cells per well, adjusted based on growth rates, and incubated overnight at 37 °C with 5% CO_2_. Experimental wells were treated with yaequinolone derivatives at concentrations of 0.4, 2, 10, 20, and 50 μM, while control wells received equivalent dilutions of DMSO to minimize effects. Each group was prepared in five replicates and incubated for 72 h. After removing the drug-containing medium, 100 μL of MTT solution (5 mg/mL) was added to each well and incubated at 37 °C for 4 h. The solution was then aspirated, replaced with 200 μL of DMSO, incubated for 10 min at 37 °C, and shaken before measuring absorbance (OD) at 570 nm using a microplate reader. The tumor cell survival rate was calculated as follows: survival rate (%) = [(OD experimental group − OD blank group)/(OD control group − OD blank group)] × 100%. All experiments were conducted in triplicate.

### 3.6. Colony Formation Assay

Cells in the logarithmic growth phase were seeded at 1000 per well in 6-well plates and incubated overnight at 37 °C with 5% CO_2_. CHNQD-02792 (2.3, 4.5, and 9 μM) was added to the experimental group, while the control group received an equal dilution of DMSO. The medium color was monitored regularly, and the medium was replaced simultaneously in both groups. After 14 days, clonal clusters (approximately 50 cells) were formed. The cells were washed with PBS, fixed with 4% paraformaldehyde for 1 h, stained with crystal violet for 30 min, washed again with PBS, dried, photographed, and analyzed using Image J software (v 1.8.0.) to count colonies. The cell proliferation rate was calculated by comparing the experimental group with the control group treated with DMSO.

### 3.7. Cell Cycle Assay

Cells in the logarithmic growth phase were seeded at 350,000 cells per well in 6-well plates and incubated overnight at 37 °C with 5% CO_2_. CHNQD-02792 (2.3, 4.5, 9 μM) was added to the experimental group, while an equal dilution of DMSO was added to the control group. After 48 h, cells were collected, centrifuged at 300× *g* for 5 min at 4 °C, and resuspended in 1 mL of ice-cold PBS. This step was repeated, and the supernatant was discarded. The cells were then resuspended in 300 μL of PBS, and 700 μL of pre-cooled anhydrous ethanol was added dropwise for fixation at 4 °C overnight. Within 12–24 h, cells were centrifuged at 1000× *g* for 5 min, and the supernatant was discarded. The cells were washed with 1 mL of pre-cooled PBS, centrifuged again, and resuspended in 500 μL of PBS. RNase A and PI were then added to each tube, and the samples were incubated at 4 °C in the dark for minutes. The cells were incubated at 4 °C with RNase A and PI in the dark for 30 min. Data were collected using a flow cytometer (488 nm laser excitation, 620/29 nm filter), and results were analyzed using FlowJo software (v.10.8.1).

### 3.8. Apoptosis Assay

Cells in the logarithmic growth phase were seeded at 600,000 cells per well in 6-well plates and incubated overnight at 37 °C in a 5% CO_2_ incubator. CHNQD-02792 (2.3, 4.5, 9 μM) was added to the experimental group, while the control group received an equal dilution of DMSO. After 48 h, cells were collected, centrifuged at 300× *g* for 5 min, and washed once with PBS. The cells were gently resuspended and counted. Take 1–5 × 10^5^ resuspended cells, centrifuge at 300× *g* for 5 min, and discard the supernatant. The cells were washed once with PBS, centrifuged again, and the supernatant was discarded. Then, 500 μL of 1× diluted Annexin V Binding Buffer was added to resuspend the cells. Add 5 μL of Annexin V-FITC and 5 μL of PI (50 μg/mL) to the cell suspension. After gently vortexing, incubate the samples at room temperature in the dark for 15–20 min. Immediately analyze the samples using a flow cytometer. The results were analyzed using Novoexpress software (v.1.6.2).

### 3.9. Western Blot Assay

Cells in the logarithmic growth phase were seeded at 600,000 cells per well in 6-well plates and incubated overnight at 37 °C with 5% CO_2_. CHNQD-02792 (2.3, 4.5, and 9 μM) was added to the experimental group, while an equivalent dilution of DMSO was added to the control group. After 24 h of treatment, the cells were collected from the incubator. The cells were washed twice with pre-cooled PBS, and the proteins were extracted using ice-cold RIPA lysis buffer (RIPA-PMSF, 99:1, *v*/*v*). Adherent cells were scraped off the Petri dish with a pre-cooled plastic scraper. Protein concentrations were measured using a BCA kit. Protein samples were mixed with 5× loading buffer, boiled in a metal bath for 6 min, and stored at −20 °C. Proteins of different molecular weights were separated by gel electrophoresis and transferred onto PVDF membranes using wet transfer. The membrane was blocked with 5% skimmed milk powder for 2 h, followed by incubation with the primary antibody overnight at 4 °C. After three washes with TBST (10 min each), the membrane was incubated with the secondary antibody at room temperature for 1 h. It was then washed three times with TBST (10 min each) and analyzed using a gel imaging system with a high-sensitivity ECL luminescent solution.

### 3.10. Molecular Docking

The MOE 2022.2 software was employed to conduct molecular docking experiments. The X-ray crystal structures of CDK1 (PDB: 3QX3, 2.16 Å), ERK (PDB: 4QTB, 1.40 Å), and JNK (PDB: 5WGG, 2.04 Å) proteins were retrieved from the Protein Data Bank (PDB). The resulting postures from the docking simulations were visualized and analyzed using Pymol (version 2.4).

### 3.11. Statistical Analysis

Experimental data were statistically analyzed using Graph Prism.10. */^ *p* < 0.05 was considered significant, **/^^ *p* < 0.01 highly significant, ***/^^^ *p* < 0.001 very highly significant, ****/^^^^ *p* < 0.0001 extremely significant. All experiments were performed in triplicate.

## 4. Conclusions

CHNQD-02792 exhibited strong cell selectivity, showing potent cytotoxicity against HT-29 cells (IC_50_ = 4.5 μM), while its well-tolerated concentration in macrophages was 5.92-fold higher than that in tumor cells. CHNQD-02792 downregulated CDK1 expression, inducing G2/M phase arrest; suppressed the anti-apoptotic protein Bcl-2 while promoting the expression of pro-apoptotic proteins Caspase-3 and Bax; inhibited ERK phosphorylation; and enhanced JNK phosphorylation, ultimately leading to apoptosis in HT-29 cells. In summary, CHNQD-02792 targeted the MAPK signaling pathway in colorectal cancer cells (HT-29), causing G2/M phase arrest and subsequently inducing tumor cell apoptosis. These findings suggest that CHNQD-02792 has the potential as a highly selective and antitumor agent.

## Figures and Tables

**Table 1:** Chemical structures of derivatives of yaequinolones (**1**–**13**).

Compounds	3″*R*/*S*	R_1_	R_2_	R_3_
**1**	*R*	H	H	H
**2**	*S*	H	H	H
**3**	*R*	OCH_3_	H	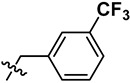
**4**	*R*	OCH_3_	H	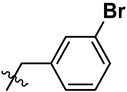
**5**	*R*	OCH_3_	H	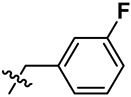
**6**	*S*	OCH_3_	H	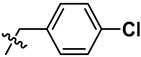
**7**	*S*	OCH_3_	H	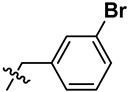
**8**	*S*	OCH_3_	H	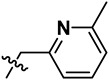
**9**	*S*	OCH_3_	H	H
**10**	*R*	H	NO_2_	H
**11**	*S*	H	NO_2_	H
**12**	*S*	Br	H	H
**13**	*R*	Br	H	H

**Table 2:** IC_50_ values in various colorectal cell lines (μM).

Compounds	CT-26	RKO	LoVo	HT-29	HCT-116	DLD-1	LS-174T
**4**	>50.0	48.3	>50.0	>50.0	>50.0	>50.0	>50.0
**7**	>50.0	22.0	25.0	>50.0	>50.0	>50.0	>50.0
**12**	26.3	37.3	>50.0	42.8	25.8	11.6	7.7
**13**	>50.0	28.0	28.29	4.5	50.0	>50.0	24.3
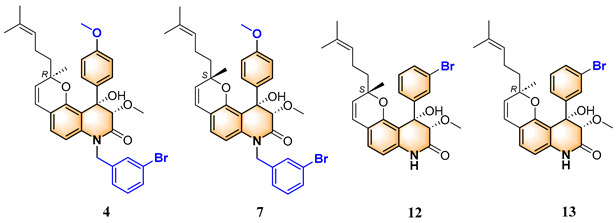							

Note: The IC_50_ value for HT-29 inhibition by the positive drug 5-fluorouracil is 15.58 μM.

## Data Availability

The data are contained within the article.

## Abbreviations

MAPK	mitogen-activated protein kinase
ERK	extracellular signal-regulated kinase
JNK	c-Jun N-terminal kinase
ASMR	age-standardized mortality rate
G2/M phase	G2 phase to M phase transition
CDK1	Cyclin-dependent kinase 1
Bcl-2	B-cell lymphoma 2
Bax	Bcl-2 associated X-protein
